# Validation of a Turkish Version of the Athlete Food Choice Questionnaire

**DOI:** 10.3390/nu15163612

**Published:** 2023-08-17

**Authors:** Yonca Sevim, Rachael L. Thurecht, Fiona E. Pelly

**Affiliations:** 1 Department of Nutrition and Dietetics, Faculty of Health Science, Bahçeşehir University, Istanbul 34353, Türkiye; 2 School of Health, University of the Sunshine Coast, Sunshine Coast 4556, Australia; fpelly@usc.edu.au

**Keywords:** diet, sport, determinant, survey, translation, Türkiye

## Abstract

There are multiple influences on food choice for athletes. The aim of this study was to assess the validity and reliability of a Turkish Athlete Food Choice Questionnaire (Turkish-AFCQ) and describe the main factors influencing food choices. A multi-step process of language and content validation, Explanatory Factor Analysis, Confirmatory Factor Analysis, and test–retest reliability were used to examine factorial structure and construct validity (convergent and discriminant) and reliability (internal and external). The translated Turkish-AFCQ was administered to 446 athletes (59% male, median age = 21 years) from a variety of sports. The original nine-factor structure was validated, external reliability was acceptable, and all factors achieved acceptable discriminate validity. Convergent validity and internal reliability received tenable-ideal ratings for seven and eight factors, respectively. Interpretation and future application are discussed for low-performing factors ‘food and health awareness’ and ‘influence of others’. The factor most frequently (never 1—always 5) influencing choices was ‘performance’ (Md = 4.33) and the least was both the ‘influence of others’ and ‘food values and beliefs’ (Md = 2.67). The Turkish-AFCQ can be used to expand researchers’ and practitioners’ understanding of the relative influence multiple factors have on food choices, and this study provides a model for AFCQ linguistic translation and validation.

## 1. Introduction

Nutrition is a critical component for enhancing performance, facilitating physiological adaptations, and preventing injury and illness in high performance athletes. Yet, athletes may struggle with adherence to dietary recommendations [1,2,3] and select foods that do not meet their training and performance needs [4]. 

Athletes are a unique population, due to sporting- and performance-related influences (such as stage of competitive season, fuelling and recovery goals, weight modification, and gastrointestinal issues) impacting on their food choices [5,6,7]. Measuring both dietary intake and determinants of food choice needs to be specific to an individual population and culture, as language interpretation and the food environment have nuances that can vary across countries. Research on the dietary intake of 334 elite Turkish athletes from 18 sports showed that their nutritional knowledge and dietary intake were not at the desired level despite being national-level athletes with generally higher levels of education [8]. The Turkish version the original Food Choice Questionnaire (FCQ) [9] targeted at general populations was validated to help explain dietary intake in Turkish populations through determinants of food choice. Application of the Turkish FCQ with 184 recreational Turkish athletes demonstrated that the factors of ‘health’, ‘natural content’, and ‘convenience’ were the most important influences on their food choices [10]. However, a limitation of using the FCQ with athletes was that the questionnaire did not encompass the sporting and performance dimensions relevant to athletes, especially those at professional and elite levels. 

To explore determinants of food choice in English-speaking populations, the Athlete Food Choice Questionnaire (AFCQ) was developed using a 5 points scale (1 never to 5 always) [11,12]. The AFCQ consisted of 32 items representing nine factors that included nutritional attributes of the food, emotional influences, food and health awareness, influence of others, usual eating practices, weight control, food values and beliefs, sensory appeal, and performance. In addition, 13 simple factors (single-item constructs) representing influences such as hunger, gut comfort, doping concern, cost, availability, and convenience have been recommended for inclusion in studies exploring determinants of food choice in athletes. The factorial structure, construct validity, and reliability of the AFCQ have subsequently been assessed in independent samples of international high-performance athletes [12,13]. 

A recent systematic review of international applications of the original FCQ [14] highlighted the cross-cultural interest in understanding food choices and importance of thorough translation and adaption of the questionnaire for different cultures [15]. Although the AFCQ development and validation work was conducted with athletes from diverse sports and countries, linguistic and cultural nuances are known to impact the validity and reliability of health behaviour questionnaires, thus translating the AFCQ into another language requires explicit assessment [16]. Therefore, the aim of this study was to (1) describe the linguistic and cultural adaptation of the 32-item AFCQ and 13 additional single items from English into Turkish, and (2) assess the validity and reliability of the nine-factor AFCQ. A secondary aim was to report on the descriptive results of the factors that most frequently influenced Turkish athletes’ food choices.

## 2. Materials and Methods

**Figure 1:**
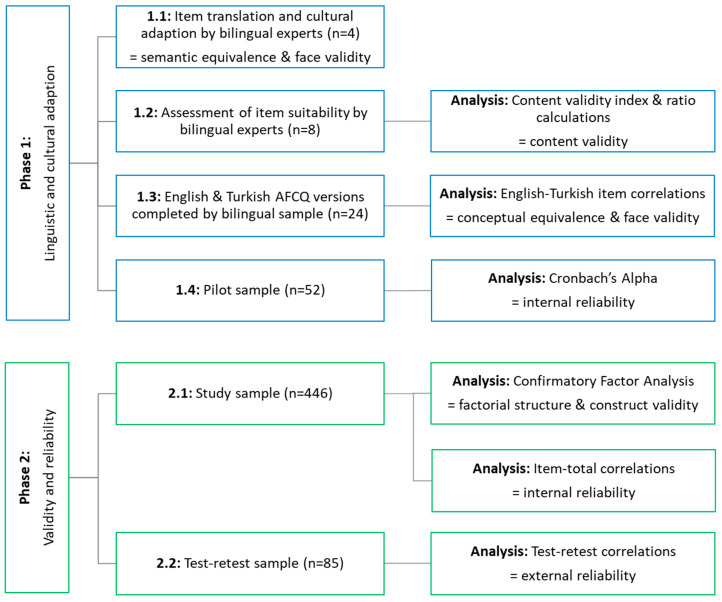
Study outline showing the samples, analyses, and purposes for each component.

In this validation study, multiple steps were undertaken to translate, culturally adapt, validate, and assess the reliability of a Turkish-AFCQ (Figure 1). The 45-question items were presented as neutral statements, where participants rated the frequency (1 never to 5 always) that each item had on their usual food choices. Food choices in the AFCQ have been defined as a single meal or an individual food or drink (beverage). 

### 2.1. Phase 1: Language Validity 

A multi-step process utilising forward translation and testing with mono and bi-lingual participants was used for testing linguistic equivalence [17]. 

Step 1: Translation from English into Turkish was completed independently by a formal translator and three subject matter experts. The translations were considered against the original and Turkish FCQ. The expert group complied and deliberated the translated the items for cultural appropriateness and semantic meaning until a consensus was reached. 

Step 2: Bilingual experts (*n* = 8) in food choice, sports nutrition, and Turkish language subject areas examined the content’s validity by rating the suitability of each question item on a 4-point Likert-type scale (1: not suitable, 2: somewhat appropriate, 3: quite appropriate, and 4: extremely appropriate). Responses were examined for the Content Validity Index (CVI) of the whole questionnaire and Content Validity Ratio (CVR) for each item [18]. The acceptable thresholds used for content validity were CVI > 0.7 and CVR > 0.0.

Step 3: The English and Turkish questionnaires were completed one day apart by bilingual athletes, dietitians, and dietetic students (*n* = 24) to examine conceptual equivalence, as indicated by Spearman Rank Correlations (r_s_) between versions. 

Step 4: The translated items were piloted with a sample of Turkish athletes (*n* = 51) to screen for an initially acceptable level of internal reliability indicated by Cronbach’s Alpha (CA) ≥ 0.7 [19].

### 2.2. Phase 2: Validity and Reliability 

The data collection tool for phase 2 of the study contained two parts: part one captured the participant’s socio-demographic characteristics, such as sex, age, income, sport, religion, and education, and part two consisted of the 32-item AFCQ and 13 additional single items. The validity and reliability testing using factor analysis was conducted on the 32-item AFCQ. 

Athletes who were healthy adults between the ages of 18 and 65 who played sport on a recreational, amateur, professional, or elite level were invited to complete the final survey. Individuals were excluded if they had not participated in any sport for more than 3 months.

A convenience sample of participants were recruited in-person and online by the lead researcher, who shared the questionnaire with sports clubs, athlete health centres, and sports departments of universities in Türkiye between January and August 2022. Participation was voluntary, and all participants were informed in detail about the study and provided written consent. The study was conducted in accordance with the Declaration of Helsinki and approved by the Institutional Ethics Committee of Bahçeşehir University Ethics Committee (#2021/10).

A target sample of 320 was set to achieve the recommended 10:1 participant-to-item ratio for factor analysis [20]. To measure the invariance of the Turkish-AFCQ over time, a test–retest application was applied to participants within a two-week interval. 

### 2.3. Data Analysis 

The Statistical Package for the Social Sciences (SPSS) version 26.0 and IBM SPSS AMOS version 25.0 (AMOS software; IBM Corp., Armonk, NY, USA) were used for statistical analysis. Significance was set at α = 0.05, correlations (r), and median (Md). Exploratory Factor Analysis (EFA) and Confirmatory Factor Analysis (CFA) were applied in study phase 2 to examine the factorial structure and construct validity. Exploratory Factor Analysis is a multivariate statistic that aims to find and discover a small number of unrelated and conceptually meaningful new variables (factors and dimensions) by bringing together interrelated variables [21,22]. The suitability of the data for factor analysis was tested using Kaiser–Meyer–Olkin (KMO) sampling adequacy (acceptable threshold 0.70) and a significant result for the Bartlett’s Test of Sphericity [21]. Items were considered for removal if the variance was <0.20, and the anti-image matrix diagonal value was <0.50. The CFA examined the fit of the data to the factorial structure (model) and was conducted using AMOS 25.0 program. Modification index and model fit indices (general-model-, relative-, absolute-, and noncentrality based) were used to examine the goodness-of-fit and if model adjustments were needed. Thresholds for an ‘acceptable’ and ‘good’ fit were presented alongside each result. 

Measures of convergent and discriminant validity were evaluated for each factor to support the construct validity of the Turkish-AFCQ. Convergent validity was acceptable with factor loadings and the average variance extracted (AVE) scoring ≥ 0.50 [21]. Discriminant validity was acceptable if the square root of the AVE for each factor was greater than the inter-factor correlations [21,23]. Internal consistency was examined through composite reliability (CR) and Cronbach’s Alpha scores for each factor, each item’s impact on Cronbach’s Alpha if it were deleted, and the corrected item–total correlations (r). The Cronbach’s Alpha was reported with a 95% CI [24]. Pearson correlation analysis was performed for examining external validity through test–retest. A coefficient (r) between 0.70 and 1.00 indicated a high degree of correlation. 

Nine AFCQ factors and 13 simple factors were reported for Md score and examined according to sex and nutrition education. Certain factors were examined in more detail based on outcomes from a previous study [13]. This included items within the ‘food values and beliefs’ factor, which were examined against religious category (two categories: Muslim, other—Christian and not religious) and strength of belief (1: not religious to 5: very religious). Similarly, items within the ‘influence of others’ and ‘food and health awareness’ factors were examined against living situation (four categories: alone, parents, family/partner, and roommate).

## 3. Results

### 3.1. Phase 1: Language Validity

Step 1: The questionnaire was translated from English to Turkish by a formal translator and three subject matter experts. The item containing examples ‘My cultural style of eating (e.g., S. American, Indian, Western)’, was changed to ‘My cultural style of eating (e.g., Aegean, Black Sea, Southeastern Anatolia, etc.)’ for greater relevance to Turkish cuisine. 

Step 2: The CVI value of the Turkish-AFCQ was obtained as 0.96, and all items had CVR scores >0 affirming content validity, indicating that no item needed eliminating.

Step 3: A sample of 24 bilingual individuals comprising athletes, dietitians, and dietetic students completed both versions of the AFCQ. The scores obtained from the English and Turkish versions were found to be significantly correlated (r_s_ = 0.85, *p* < 0.001), indicating conceptual similarity between versions. 

Step 4: The 32-item Turkish-AFCQ was pilot tested on 51 athletes to examine initial internal consistency. The Cronbach’s Alpha values for each factor were acceptable: ‘nutritional attributes of the food’ CA = 0.89, ‘emotional influences’ CA = 0.90, ‘food and health awareness’ CA = 0.90, ‘influence of others’ CA = 0.89, ‘usual eating practices’ CA = 0.89, ‘weight control’ CA = 0.90, ‘food values and beliefs’ CA = 0.90, ‘sensory appeal’ CA = 0.89, and ‘performance’ CA = 0.89.

### 3.2. Phase 2: Validity and Reliability 

A sample of 446 athletes from a variety of sports who were identified in the recreational (21%), amateur (48%), or professional–elite (31%) levels participated in the validation phase. The participant-to-item ratio was 14:1, exceeding the desired 10:1 guideline for factorial analysis. The sample included 268 males (59%) and 178 females (31%) between the ages of 18 and 61 years (Md = 21 and IQR = 18–25 years). Participants reported exercising on average 5 days per week (range: 1–7 days) and had an average length of participation in sports of Md = 8 years (range: 1–30 years). Demographic characteristics shown in Table 1. 

#### 3.2.1. Explanatory Factor Analysis

The data were suitable for EFA, as indicated by a KMO sampling adequacy score of 0.92 and a significant Bartlett’s Test of Sphericity (χ^2^ = 8483.24, *p* < 0.001). In the factor structure, a structure of nine factors was determined, which explained 77.0% of the total variance. As nine sub-dimensions explained 69.6% of the variation in the total variance, the explanation rate of the factors was found to be sufficient, and variance for each factor is shown in Table 2. All 32 items exceeded the variance and anti-image matrix thresholds; therefore, no items were removed. The EFA factor loadings varied between 0.54 and 0.87, with 20 exceeding the ideal threshold of 0.70 [20]. 

#### 3.2.2. Confirmatory Factor Analysis

The modification index showed no need for any variation in the model. The ratio value between the chi-square statistic and degree of freedom was 2.12, indicating a good fit, whereas the other fit indices were either good or acceptable (Table 3). Under these parameters, the construct validity of the factorial structure was confirmed. In support of convergent validity, (1) factor loadings were ideal for 19 items, acceptable for 9, and unacceptable for 4 items (Table 2), and (2) six factors satisfied the AVE threshold (Table 4). Discriminant validity was deemed acceptable for all factors, as their square root of the AVE was greater than their inter-factor correlations (Table 4).

#### 3.2.3. Reliability

Four factors received ideal composite reliability scores, with another four being acceptable and one unacceptable (Table 2). The Cronbach’s Alpha scores ranged from 0.52 to 0.89 (Table 2), with four factors achieving acceptable or higher scores, two were tolerable, and three received unacceptable scores (<0.60). Item–total correlations varied between 0.28 and 0.84, with 50% achieving good correlation levels ≥ 0.50, and only one item correlating below the acceptable 0.3 threshold. Interitem correlations within the respective factors ranged from 0.17 to 0.84.

Three factors contained an item that was correlated at <0.3 with the other indicator items of the factor. The items were ‘my ability to cook for myself’ (factor: food and health awareness), ‘my cultural style of eating (e.g., Aegean, Black Sea, Southeastern Anatolia)’ (factor: usual eating practices), and ‘whether I am in the off season (no competitions or intense training for a period of time)’ (factor: weight control).

Test–retest analysis was conducted on a subsample of 85 athletes. A significant positive correlation was found at the level of r = 0.78 and *p* < 0.01 between the total scores of the two tests. In this context, it was determined that the results of the Turkish-AFCQ did not change depending on time and had satisfied this measure of external reliability. A summary of the validity and reliability interpretations is given in Table 5 to show where there is agreement among the psychometric parameters examined in this study. 

#### 3.2.4. Descriptive Outcomes

The factor most frequently influencing athlete food choices was ‘performance’, whereas the least frequent were both the ‘influence of others’ and ‘food values and beliefs’ (Table 6). Females were more frequently influenced by ‘emotional influences’ (Md = 3.00 verses Md = 2.25, U = 14,054.0, *p* < 0.001) and ‘sensory appeal’ (Md = 4.00 verses Md = 3.67, U = 18,346.5, *p* < 0.001). Males were more frequently influenced by ‘weight control’ (Md = 3.75 verses Md = 3.50, U = 28,499.5, *p* < 0.001). 

**Figure 2:**
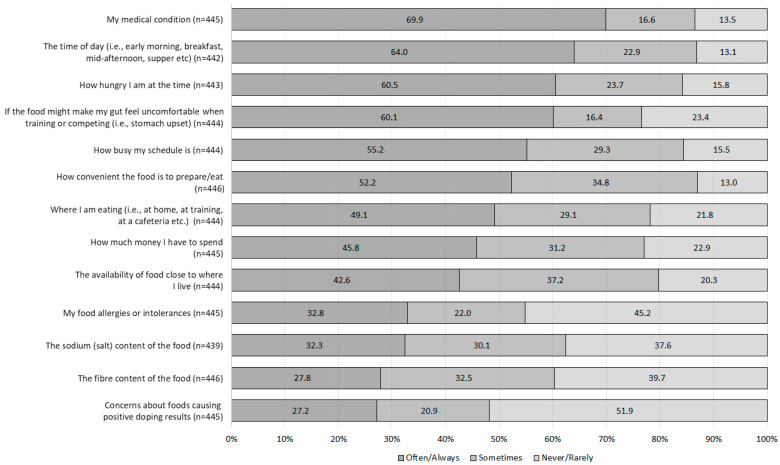
The percentage of athletes who rated how frequently (never/rarely, sometimes, and often/always) 13 items influenced their food choices.

Across the 13 optional items, medical condition, time of day, and hunger were the three items receiving the greatest proportion of often/always responses, whereas doping concerns, fibre content, and sodium content were the three receiving the greatest rarely/never responses (Figure 2). Extent of religious belief and religion were significantly related with Muslim participants having stronger extent of beliefs compared to those from other religions (moderately religious *n* = 149, 99.3% Muslim; very religious *n* = 52, 100% Muslim; and X^2^(3) = 134.4, *p* < 0.001). The ‘food values and beliefs’ factor was rated as a more frequent influence by Muslim participants (Md = 2.67) than those identifying in the other/non-religious category (Md = 1.67; U = 5872.0, *p* < 0.001). The ‘my religious food beliefs’ and ‘if the food is sustainably produced’ items of the factor ‘food values and beliefs’ received a greater proportion of often (*n* = 48, 98.0%; *n* = 79, 90.8%) and always (*n* = 117, 98.3%; *n* = 39, 97.5%) responses by Muslim participants compared to other/non-religious participants (X^2^(4) = 84.2, *p* < 0.00; X^2^(4) = 14.4, *p* = 0.006), respectively. No significant differences detected among items in the ‘influence of others’ or ‘food and health awareness’ factors according to participant living situation.

## 4. Discussion

This study describes the translation and cultural adaptation of the AFCQ from English into Turkish and provides evidence of validity and reliability to inform the application of this translated version to Turkish athletes. The EFA and CFA results for the Turkish-AFCQ demonstrated that the fit for the 9-factor model confirmed the model’s structure. The most frequently influential factors of ‘performance’, ‘food and health awareness’, and ‘sensory appeal’ and the least of ‘emotional influence’, ‘influence of others’, and ‘food values and beliefs’ were the same as reported in previous applications of the AFCQ [13,25]. 

Overall, there is consistent evidence to support the construct validity of the factors ‘nutritional attributes of the food’, ‘performance’, ‘weight control, ‘usual eating practices’, and ‘food values and beliefs’. A further two factors, ‘emotional influences’ and ‘sensory appeal’, were considered valid, as all results for validity and reliability were acceptable, with the exception of one marginal attribute. Conversely, the two lowest-performing factors (‘food and health awareness’ and the ‘influence of others’) had more than one attribute that did not meet the acceptable threshold. 

Internal reliability results were notably different between Cronbach’s Alpha and composite reliability measures in this study. All factors except ‘influence of others’ achieved acceptable composite reliability scores for the Turkish-AFCQ. The Cronbach’s Alpha scores were tolerable or better for 7 factors with exception of ‘usual eating practices’ and ‘food values and beliefs’. This was consistent with the reliability of the original AFCQ, where the same factors received the lowest Cronbach’s Alpha scores [13]. 

On examination of the factor items, unsurprisingly, the item on religious food beliefs was more frequently an influence for Muslim religious participants. Similarly, the item on sustainability had similar proportions of Muslim religious participants rating it as often or always an influence. The descriptive results supported the previous hypothesis in the validation study of the original AFCQ that the ‘food values and beliefs’ factor retained relevance with populations from non-western regions where religious influence has a pervasive role in society [13]. The acceptable construct validity and composite reliability results of this study supported the inclusion of the ‘food values and beliefs’ factor in the Turkish-AFCQ and, furthermore, is useful in research examining sustainability and religious influences on eating behaviours. The low Cronbach’s Alpha scores may have been due to violations of tau-equivalence (i.e., equal factor loadings), which could result in a potential underestimation of the true reliability of a factor [26,27]. Therefore, we recommend to researchers using the Turkish-AFCQ that they employ Cronbach’s Alpha with another reliability measure that is more robust to unequal item variance, such as the CFA driven composite reliability score, the McDonald (1999) omega coefficient, or the Greatest Lower Bound measure [28]. 

The factors ‘food and health awareness’ and ‘influence of others’ received the lowest convergent validity results. Interestingly, these two factors had acceptable convergent validity in the initial CFA for the AFCQ [12]. The ‘food and health awareness’ factor is theoretically akin to the concept of food literacy, encompassing a person’s ‘inter-related knowledge, skills and behaviours required to plan, manage, select, prepare and eat food to meet needs’ [29]. It is feasible that there may be a multicollinearity issue among other Turkish-AFCQ factors related to food literacy or that there are insufficient indicator items to represent adequately and consistently ‘food and health awareness’. The International Food Literacy Questionnaire (IFLQ-19) developed utilising an EFA method that extracted 19 factors or components highlights the multidimensionality of this concept and the complexities of capturing the influence that ‘food and health awareness’ contributes towards food choices. Future research examining the Turkish-AFCQ and a translated IFLQ-19 could provide valuable insights into the relationship between the ‘food and health awareness’ factor and food literacy of Turkish athletes.

Examination of the inter-factor correlations shows that ‘food and health awareness’ has a low–moderate positive correlation (r = 0.43) with the factor ‘nutritional attributes of the food’. However, the correlation is not strong enough for multicollinearity to be an issue, nor is the level of correlation sufficient to explain the convergent validity results. A final consideration is that the item ‘my ability to cook for myself’, which only weakly correlated with other indicator items, was interpreted differently by participants in this sample. Given that few participants lived alone in our sample, it is possible that cooking for oneself is of less relevance; however, our analysis detected no significant differences based on living situation. Future applications of the Turkish-AFCQ would benefit from pilot testing the questionnaire with the indicator item expressed as ‘my ability to cook’, and examining item-to-item and item-to-total correlations. Thus, we recommend cautiously interpreting findings in relation to this factor and consider inter-factor correlations with the factor ‘nutritional attributes of the food’. 

The ‘influence of others’ scored the lowest among the nine Turkish-AFCQ factors. Despite acceptable discriminant validity and item-to-total correlations, both convergent validity and reliability measurements were considered insufficient. Previously, the ‘influence of others’ was found to have a lower Cronbach’s Alpha when administered to a mixed group of athletes (online sample) as compared to those who completed the AFCQ during a major competition [13]. Similarly, the current sample was collected with Turkish athletes outside of a major competition environment; therefore, the influence of family, friends, and other athletes may have been less consistent. The current study reaffirmed the previous suggestion that the social context should be considered when applying the AFCQ [13]. Interestingly, analysis did not find a significant difference in the indicator items for the factors based on living situation in this sample. However, given that influences of teammates and other impacts on athlete food choices across different cultures [7], we do not recommend omitting this factor. Therefore, future research that includes detailed questioning of the athlete’s social and living circumstances would enhance our understanding of social influences on Turkish athletes’ food choices, which could lead to refinement of the ‘influence of others’ factor. 

Prior nutrition education appeared to increase the frequency that Turkish athletes are influenced by factors such as ‘food and health awareness’, ‘nutritional attributes of the food’, ‘weight control’, and ‘usual eating practices’. These factors are relevant to topics of nutrition education and eating-behaviour-change interventions [30,31]. Given that the key services of sports nutrition professionals are providing nutrition education and tailored support surrounding current eating behaviours, the increased frequencies of these factors are logical. Application of the Turkish-AFCQ with a nutrition knowledge intervention may assist in tracking athlete awareness of educational messages. Furthermore, the Turkish-AFCQ may assist in identifying target areas for education to individual or groups of athletes based on the factors influencing their food choices. For example, a low frequency of influence from the ‘nutritional attributes of the food’ and ‘food and health awareness’ could indicate that nutritional knowledge or the importance of nutrition could be improved. Future research evaluating the utility of the Turkish-AFCQ in tailoring athlete educational interventions is recommended. 

This methodological study has some limitations that impact the generalisability of the findings. Most notably, the study sample was recruited via convenience sampling, which had the potential to introduce participant self-selection and social desirability biases. Participants may inherently have a greater interest in food and nutrition or higher education level impacting on the representativeness and generalisability of findings. Future research with a representative sampling strategy may overcome this limitation. As this study relies on self-reported data, there is risk of participants misrepresenting their food choice influences in order to provide responses favouring social norms. However, the question items strived for neutral phrasing to help mitigate social sensitivity associated with responding to the questionnaire. Application of the AFCQ of any language is recommended to be performed without the researcher present and with assuring anonymity for the participant to help them feel at ease providing truthful responses. A further limitation is that the sample is not representative of exclusively high-performance Turkish athletes. However, even though the sample consisted of mixed levels of athletes, those identifying as recreational were the minority. Due to the paucity of data on determinants of food choices for elite-level athletes in Türkiye, further studies are warranted and could benefit from a qualitative exploration of food choice determinants with using the Turkish-AFCQ as a primer to athlete interviews of focused group discussions. Despite these limitations, the study results showed strengths in the multi-step translation process, participant-to-item ratio, the examination of subtypes of construct validity, and achieving low inter-factor correlations. 

## 5. Conclusions

This study sought to provide thorough reporting of the translation and validation of a Turkish-AFCQ to support its application, modification, and interpretation. Multiple psychometric parameters were assessed, with the majority supporting the validity and reliability of the Turkish-AFCQ as a suitable tool for measuring the multifactorial influences on Turkish athletes’ food choices. The thorough translation and evaluation give confidence for conceptual equivalence of a Turkish-AFCQ and allow for comparison of findings utilising the English AFCQ and Turkish-AFCQ. In conclusion, the Turkish-AFCQ can be used to advance researchers’ and practitioners’ understanding of the relative influence multiple factors have on food choices providing greater context to nutrition knowledge and dietary intake data such, as illustrated in the previously published AFCQ practitioner guide (ref to Supplement 2: Thurecht and Pelly 2021) [12]. Applications of the Turkish-AFCQ with individuals or groups can help to inform nutrition education strategies that support the performance, recovery, and overall health of Türkiye’s high-performance athletes. 

## Figures and Tables

**Table 1:** Demographic characteristics of study sample (*n* = 446).

	*n*	%		*n*	%		*n*	%
**Sex**			**Type of Athlete**			**Nutrition Education**		
Female	178	41	Recreational	95	21	Yes—nutritionist or dietitian	70	15.7
Male	268	59	Amateur	215	48	Yes (trainer)	79	17.7
**Income**			Professional/Elite	136	31	Yes (other)	20	4.5
Income < Expenses	52	12	Sport and Exercise Categories *			Yes (studied nutrition)	12	2.7
Income = Expenses	232	52	Aesthetic	11	2.5	Yes (doctor)	6	1.3
Income > Expenses	162	36	Weight category	64	14.3	No	259	58.1
**Living Situation**			Team	213	47.8	**Religious Category**		
Alone	56	13	Racket	18	4.0	Muslim	364	82.4
Roommate/friend	32	7	Outdoor sports	7	1.6	Christian	2	0.5
Partner	10	2	Skill-based	2	0.4	Prefer not to say	14	3.2
Family (married +/− children)	32	7	Swimming and athletics	48	10.8	Not religious	62	14.0
Parent/s	316	71	Gym	83	18.6	**Strength of Religious Belief**		
**Medical/Food Related Conditions**			**Education**			Not religious	139	31.2
Diagnosed chronic disease	32	7.2	High school-Secondary	179	40	A little religious	60	13.5
Eating behaviour disorder	15	3.4	University-Bachelor	229	51	Moderately religious	151	33.9
Food allergy	20	4.5	Postgraduate	38	9	Very religious	53	11.9
Food intolerance	61	13.7				Prefer not to say	43	9.6

* Aesthetic = synchronized skating and dance; Weight category = karate/boxing/kickboxing/Muay Thai, wrestling, body building, and rowing; Team = basketball, volleyball, football, hockey, underwater hockey, and handball; Racket = tennis, squash, badminton, and table tennis; Outdoor sports = mountaineering, trekking, and karting; Skill-based = sailing and archery; Swimming and athletics = swimming, athletics, running, cycling, and triathlon; Gym = personal trainer, fitness/indoor exercises, and yoga/plates/reformer.

**Table 2:** Internal reliability, factor loading and item distinctiveness results (*n* = 446).

	EFA Variance Explained	CFA Factor Loadings	Composite Relibaility	Cronbach’s Alpha (95%CI)	CA, If Item Deleted	Item–Total Correlations
Factor 1: Nutritional attributes of the food	19.24		0.928	0.751 (0.712–0.785)		
The presence of vitamins and minerals in the food		0.88			0.680	0.585
The natural content of the food		0.91			0.672	0.618
The health or nutrition claims about the food		0.74			0.718	0.482
The nutritional content of the food (protein, fat carbohydrate)		0.99			0.708	0.514
Whether the food is a wholefood		0.70			0.753	0.411
Factor 2: Emotional influences	15.99		0.850	0.878 (0.859–0.896)		
How sad I feel		0.67			0.818	0.802
How stressed I feel		0.82			0.802	0.840
How angry I feel		0.55			0.814	0.812
Eating to comfort my emotions		0.71			0.926	0.519
Factor 3: Food and health awareness	8.892		0.710	0.665 (0.611–0.713)		
My ability to plan my foods ahead		0.32			0.587	0.460
My ability to cook for myself		0.53			0.683	0.345
My knowledge of nutritious foods		0.90			0.565	0.508
My awareness of the foods I already consumed today		0.66			0.560	0.51
Factor 4: Influence of others	6.034		0.299	0.595 (0.525–0.656)		
What other athletes in my sport are eating		0.25			0.626	0.314
What my friends are eating		0.18			0.295	0.548
What my family is eating		0.61			0.547	0.372
Factor 5: Usual eating practices	5.478		0.813	0.519 (0.436–0.592)		
How familiar the food is to me		0.96			0.422	0.334
The foods that I’ve grown up eating		0.73			0.305	0.402
My cultural style of eating (e.g., Aegean, Black Sea, Southeast Anatolia)		0.59			0.528	0.276
Factor 6: Weight control	4.102		0.852	0.662 (0.607–0.710)		
If I am trying to lose or gain weight		0.78			0.608	0.423
If the food is beneficial for my weight goal		0.65			0.522	0.555
How happy I am with my current weight/body image		0.74			0.580	0.463
Whether I am in the off season (no competitions or intense training for a period of time)		0.89			0.662	0.344
Factor 7: Food values and beliefs	3.998		0.775	0.545 (0.466–0.613)		
If the food aligns with my values for animal welfare (i.e., no animal products/vegan, cruelty-free raised animals)		0.81			0.486	0.329
My religious food beliefs		0.84			0.509	0.337
If the food is sustainably produced		0.52			0.350	0.433
Factor 8: Sensory appeal	3.103		0.767	0.713 (0.663–0.756)		
The flavour of the food		0.80			0.498	0.656
The taste of the food		0.99			0.482	0.672
The sensory appeal of available foods		0.28			0.912	0.359
Factor 9: Performance	2.998		0.744	0.891 (0.873–0.908)		
My need to fuel my body for competition		0.81			0.869	0.773
My need to feel energetic for training & competing		0.74			0.810	0.834
My need to fuel my body for recovery		0.54			0.860	0.771

EFA: Exploratory Factor Analysis, CFA: Confirmatory Factor Analysis, CA: Cronbach’s Alpha, and CI: Confidence Interval.

**Table 3:** Model fit results from the Confirmatory Factor Analysis (*n* = 446).

Fit Indices	Good	Acceptable	Result	Interpretation
General Model Fit				
Χ2/df	≤3	≤4–5	2.12	Good fit
Comparative Fit Indices				
NFI	≥0.95	0.94–0.90	0.927	Acceptable fit
NNFI	≥0.95	0.94–0.90	0.938	Acceptable fit
IFI	≥0.95	0.94–0.90	0.972	Good fit
CFI	≥0.97	≥0.95	0.980	Good fit
RMSEA	≤0.05	0.06–0.08	0.017	Good fit
Absolute Fit Indices				
GFI	≥0.90	0.89–0.85	0.945	Good fit
AGFI	≥0.90	0.89–0.85	0.967	Good fit
Residual-Based Fit Indices				
RMR	≤0.05	0.06–0.08	0.023	Good fit

df: Degrees of Freedom, NFI: Normed Fit Index, NNFI: Non-Normed Fit Index, IFI: Incremental Fit Index, CFI: Comparative Fit Index, RMSEA: Root Mean Square Error of Approximation, GFI: Goodness of Fit Index, AGFI: The Adjusted Goodness of Fit Index, and RMR: The Root Mean Square Residual.

**Table 4:** Intercorrelations between the nine food choice factors.

	Nutritional Attributes of the Food	Emotional Influences	Food and Health Awareness	Influence of Others	Usual Eating Practices	Weight Control	Food Values and Beliefs	Sensory Appeal	Perfor-Mance
Nutritional attributes of the food	**0.851**								
Emotional influences	0.048	**0.694**							
Food and health awareness	0.426	−0.066	**0.638**						
Influence of others	0.137	0.188	0.195	**0.395**					
Usual eating practices	0.319	0.105	0.300	0.345	**0.775**				
Weight control	0.402	0.063	0.321	0.179	0.199	**0.770**			
Food values and beliefs	0.131	0.169	0.112	0.219	0.186	0.075	**0.738**		
Sensory appeal	−0.023	0.353	−0.051	0.120	0.242	0.058	0.110	**0.752**	
Performance	0.352	−0.046	0.312	0.186	0.177	0.435	0.089	0.062	**0.706**
AVE	0.724	0.482	0.407	0.156	0.601	0.593	0.544	0.566	0.498

AVE = Average variance extracted. Bolded values along the diagonal display the square root of the AVE, and values below this display the inter-factor correlation matrix.

**Table 5:** Summary validity and reliability of interpretations for each factor.

	Discriminant Validity	Convergent Validity		Reliability	
Factor	√ AVE > Inter- Factor Correlation	AVE	Item Factor Loadings (# Items)	Composite Reliability	Cronbach’s Alpha
Nutritional attributes of the food	Acceptable	Acceptable	Ideal (5/5)	Good	Acceptable
Performance	Acceptable	Acceptable	Ideal (2/3), acceptable (1/3)	Acceptable	Good
Weight control	Acceptable	Acceptable	Ideal (3/4), acceptable (1/4)	Good	Tolerable
Usual eating practices	Acceptable	Acceptable	Ideal (2/3), acceptable (1/3)	Good	Unacceptable
Food values and beliefs	Acceptable	Acceptable	Ideal (2/3), acceptable (1/3)	Acceptable	Unacceptable
Sensory appeal	Acceptable	Acceptable	Ideal (2/3), unacceptable (1/3)	Acceptable	Acceptable
Emotional influences	Acceptable	Tolerable	Ideal (2/4), acceptable (2/4)	Good	Good
Food and health awareness	Acceptable	Tolerable	Ideal (1/4), acceptable (2/4), unacceptable (1/4)	Acceptable	Tolerable
Influence of others	Acceptable	Unacceptable	Acceptable (1/3), unacceptable (2/3)	Unacceptable	Tolerable

AVE = average variance extracted.

**Table 6:** Median factor scores according to sex and nutrition education for 446 athletes.

Factor		Sex		*p*	Nutrition Education		*p*
	Total	Female	Male	Value	Yes	No	Value
Performance	4.33	4.00	4.67	0.005	4.33	4.33	NS
Food and health awareness	3.75	3.75	3.50	NS	4.00	3.50	<0.001
Sensory appeal	3.67	4.00	3.67	<0.001	4.00	3.67	NS
Nutritional attributes of the food	3.60	3.60	3.60	NS	3.80	3.40	0.011
Weight control	3.50	3.50	3.75	<0.001	3.75	3.50	<0.001
Usual eating practices	3.00	3.00	3.00	NS	3.33	3.00	0.033
Emotional influence	2.75	3.00	2.25	<0.001	2.50	2.75	NS
Influence of others	2.67	3.00	2.67	NS	3.00	2.67	NS
Food values and beliefs	2.67	2.67	2.67	NS	2.67	2.67	NS

Mann–Whitney U test: Sex = Emotional influence, 14,054.0; Weight control, 28,499.5; Sensory appeal, 18,346.5; Nutrition education = Nutritional attributes of the food, 20,824.5; Usual eating practices, 21,385.5; Food and health awareness, 18,742.5; Weight control, 19,263.5. NS: Not significant (*p* > 0.05).

## Data Availability

The data presented in this study are available on request from the corresponding author. The data are not publicly available due to privacy and ethical reasons.
